# Study on the Influence of Thermoplastic Microcapsules on the Sulfate Resistance and Self-Healing Performance of Limestone Calcined Clay Cement Concrete

**DOI:** 10.3390/molecules29204797

**Published:** 2024-10-10

**Authors:** Wei Du, Lu Jiang, Quantao Liu, Wei Chen, Qingjun Ding

**Affiliations:** 1 Hubei Provincial Engineering Research Center of Industrial Detonator Intelligent Assembly, Wuhan Textile University, Wuhan 430073, China; duwei@wtu.edu.cn (W.D.); jl0107023@163.com (L.J.); 2 School of Materials Science and Engineering, Wuhan Textile University, Wuhan 430200, China; 3 State Key Laboratory of Silicate Materials for Architectures, Wuhan University of Technology, Wuhan 430070, China; liuqt@whut.edu.cn

**Keywords:** limestone calcined clay cement (LC3), low-carbon, self-healing, microcapsules, sulfate dry–wet cycle

## Abstract

Limestone calcined clay cement (LC3), enhanced through reactions with volcanic ash and the interaction between limestone and clay, significantly improves the performance of cementitious materials. It has the potential to cut CO_2_ emissions by up to 30% and energy consumption in cement manufacture by 15% to 20%, providing a promising prospect for the large-scale production of low-carbon cement with a lower environmental effect. To effectively manufacture LC3 concrete, this study utilized limestone (15%), calcined clay (30%), and gypsum (5%) as supplementary cementitious materials (SCMs), replacing 50% of ordinary Portland cement (OPC). However, in regions abundant in sulfate, sulfate attack can cause interior cracking of concrete, reducing the longevity of the building. To address this issue, microcapsules containing microcrystalline wax, ceresine wax, and nano-CaCO_3_ encapsulated in epoxy resin were prepared and successfully incorporated into LC3 concrete. Sulfate resistance tests were conducted through sulfate dry–wet cycles, comparing samples with and without microcapsules. The findings revealed that the initial mechanical and permeability properties of LC3 concrete did not significantly differ from OPC concrete. LC3 concrete with added microcapsules (SP4) exhibited enhanced resistance to sulfate attack, reducing mass loss and compressive strength degradation. SEM images displayed a mesh-like structure of repair products in SP4. After 14 days of self-repair, SP4 exhibited a 44.2% harmful pore ratio, 98.1% compressive strength retention, 88.7% chloride ion diffusion coefficient retention, 91.12 mV maximum amplitude, and 9.14 mV maximum frequency amplitude. The experimental results indicate that the presence of microcapsules enhances the sulfate attack self-healing performance of LC3 concrete.

## 1. Introduction

Concrete production has reached a massive scale, with a global per capita annual output of four tons. However, this scale of development is not without its costs. Cement, serving as the binder for concrete, contributes to 8% of the world’s total emissions [1]. Over the years, significant progress has been achieved in the cement industry’s energy-saving and emission reduction efforts, especially with the widespread adoption of new dry cement production technologies and the substantial use of supplementary cementitious materials (SCMs) such as slag and fly ash. This has caused the proportion of clinker per ton of cement to drop and a decrease in CO_2_ emissions [2].

Nevertheless, CO_2_ emissions from cement manufacturing still constitute 7% of the global total, making it crucial for achieving global carbon reduction goals. The advancement of carbon emission trading will profoundly impact the global cement industry [3]. Clinker, a primary component of cement, is responsible for an extremely high carbon footprint during its production, accounting for 90% of the total emissions in the concrete manufacturing process. Tan [4] explored the properties of cement mortar containing pulverized biochar pyrolyzed at different temperatures, suggesting a potential alternative to traditional cement production methods. Iakovleva [5] discussed the benefits of renewable energy, such as solar power plants, in reducing carbon dioxide emissions in Cuba. Barría [6] studied the microstructure and mechanical behavior of cement modified with a biopolymer after carbonation, aiming to analyze the poromechanical behavior of cement in a CO_2_ environment. Jani [7] proposed Class C fly ash-based alkali activated cement as a potential alternative for CO_2_ storage applications, offering a new approach to reducing carbon emissions in the cement industry. Sadok [8] evaluated the effects of adding calcined canal sediments and ground granulated blast furnace slag (GGBS) to cement mixtures to reduce carbon footprint and cost. Their study focused on the mechanical properties and microstructure of low carbon binders, providing insights into sustainable cement production practices.

In the international roadmap for energy-saving and environmentally friendly development in cement manufacturing, the development and widespread application of high-performance SCMs, as substantial substitutes for cement and clinker, are recognized as one of the most critical technological pathways for achieving low-carbon development in the cement and concrete industry [9]. Statistical predictions indicate that this technology is expected to contribute to a 37% reduction in total carbon emissions from the cement industry by 2050. Fortunately, there are numerous effective strategies to significantly reduce the amount of clinker required for cement and concrete manufacturing. One of the most promising solutions is known as LC3, or limestone calcined clay cement [10].

LC3 originated from research conducted at the EPFL and is a kind of cement composed of limestone, calcined clay, gypsum, and clinker [11]. In LC3, the interaction of limestone and calcined clay produces carbonated aluminosilicate hydrates, which aid in cement hydration. The hydration products rise as the amount of calcined clay increases. Thus, even at relatively high replacement rates of cement clinker, LC3 maintains higher mechanical performance [12,13]. Compared to the primary SCMs, fly ash, and slag, widely used in current blended Portland cements, calcined clay [14] is considered a promising material for the future due to two main reasons. For starters, the supply of fly ash and slag is restricted in many locations or countries, particularly in developing countries, limiting their broad use. In contrast, clay is globally abundant and exhibits significant potential as a SCM. Secondly, when calcined clay is compared to fly ash and slag, its chemical composition changes very little. As a result, products containing calcined clay behave more predictably and controllably [15,16,17]. According to studies, the maximum replacement percentage of calcined clay in mixed cements with calcined clay is roughly 30% without impairing performance. However, the LC3 system allows for higher levels of clinker replacement, reaching 50% or more [18], and is considered more environmentally friendly and cost competitive.

**Figure 1:**
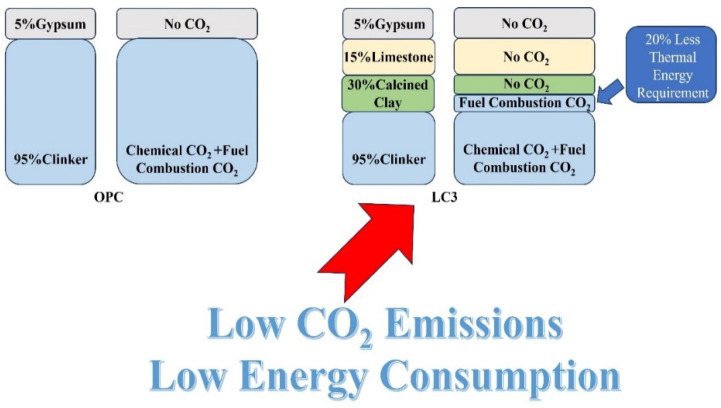
Differences in CO_2_ emissions during the manufacturing of OPC and LC3.

LC3 has effectively addressed two sources of carbon emissions in the clinker production process. Firstly, calcined clay and limestone powder are used to replace half of the clinker. LC3 avoids carbon emissions during heating, a phenomenon associated with limestone. Secondly, the lower heating temperature of clay reduces fuel consumption and, consequently, carbon emissions. Due to the relatively lower required temperature, the adoption of clean energy sources, such as electricity, becomes more feasible. In comparison to traditional cement, LC3 reduces approximately 40% of CO_2_ emissions by substituting half of the clinker. For every ton of calcined clay produced, there is a reduction of 600 kg of carbon dioxide emissions. It is estimated that by the end of 2023, LC3 decreased CO_2_ by around 15 million tons, with a projected decrease of up to 45 million tons of CO_2_ by 2025. Additionally, LC3 requires less energy for production, and the clay used is widely available, resulting in lower production costs and potentially up to a 25% reduction. Figure 1 illustrates the difference in carbon emissions during the manufacturing process between Ordinary Portland Cement (OPC) and LC3.

Concrete, as a widely used foundational material in construction engineering, must meet diverse requirements in different engineering fields, especially ensuring its structural safety performance throughout its entire lifecycle. The deteriorating process of sulfate attack affects the durability of concrete components, especially when they are exposed to sulfate-rich environments [19,20]. The fundamental mechanism of sulfate attack on concrete is the interaction of sulfate ions with hydrated products; this causes ettringite and gypsum to develop, causing concrete expansion and cracking, ultimately accelerating the collapse of buildings. Additionally, recrystallization pressure caused by sulfate in the pores leads to cracking during the concrete’s dry–wet cycling [21,22,23]. Currently, to enhance concrete’s resistance to sulfate attack, commonly employed measures include the addition of admixtures such as metakaolin, silica fume, blast furnace slag, carbon nanotubes, etc., to partially replace cement [24,25,26,27]. These admixtures, however, only serve to slow the degradation of concrete [27,28,29,30]. Once damaged in a sulfate-rich environment, concrete’s degradation rate accelerates, severely lowering its service life. As a result, improving concrete’s self-healing characteristics is critical for extending its service life in sulfate-rich environment.

The microcapsule self-healing technology, characterized by strong environmental adaptability and rapid healing, possesses significant advantages in effectively repairing microscopic cracks within concrete, demonstrating extensive application prospects [31]. The healing agent is pre-encapsulated in a shell material in this technology, and the microcapsules are subsequently combined with a curing agent within the concrete. When fractures appear throughout the service life of the concrete, tension at the crack tips causes the microcapsules to burst, allowing the healing agent to enter the crack. At the location of the crack, the healing agent reacts chemically with the curing agent within the concrete, forming healing products, thereby achieving the self-heal of the crack. In the long term, the use of microcapsules can reduce the consumption of cement; it has important implications for lowering cement production’s energy usage and CO_2_ emissions [32]. At present, thermosetting materials (urea formaldehyde [33], polyurethane [34], and melamine formaldehyde resin (MF) [35]) are commonly used as shell materials for microcapsules, which have high strength [36]. When microcracks appear in concrete, the thermosetting shell is difficult to rupture and release healing agents. Some researchers have attempted to solve this problem by using thermoplastic materials as shell materials. In the previous work, paraffin microcapsules containing toluene diisocyanate (TDI) have been prepared and applied to the self-healing of concrete [37]. However, although these microcapsules are more prone to rupture under external forces, the mechanical properties of the shell material are relatively low, and the risk of rupture during concrete mixing is high. In order to improve the strength of microcapsules, Krupa [38,39] attempted to prepare the shell material of microcapsules by mixing thermoplastic materials paraffin, polyethylene (PE), and polypropylene, while Karkri [40] used a high-density mixture of PE and MF to prepare the shell material. Furthermore, research on the resistance of concrete containing microcapsules to sulfate attack is restricted, providing a barrier to the progress and implementation of concrete with microcapsule self-healing characteristics. Additionally, the widespread application of microcapsule self-healing technology is predominantly concentrated in ordinary Portland cement concrete, with relatively limited research in the field of low-carbon cement (LC3) concrete.

To address the current limitations in research, this study employed microcrystalline wax, ceresine wax, and nano-CaCO_3_ to prepare a composite shell for encapsulating epoxy resin, forming microcapsules. Low-carbon concrete was prepared by substituting LC3 for 50% of conventional Portland cement. Subsequently, a comprehensive characterization was conducted on the concrete exposed to sulfate salt dry–wet cycles and self-healing. Mass loss, pore size distribution, microstructure, mechanical properties, permeability, and ultrasonic waveforms were all considered in this assessment. We investigated the impact of microcapsules on the concrete’s resistance to sulfate salt erosion and self-healing performance. Cracks were prefabricated on the concrete surface to examine the self-healing performance of microcapsules.

## 2. Results and Discussion

### 2.1. Mass Loss Rate

**Figure 2:**
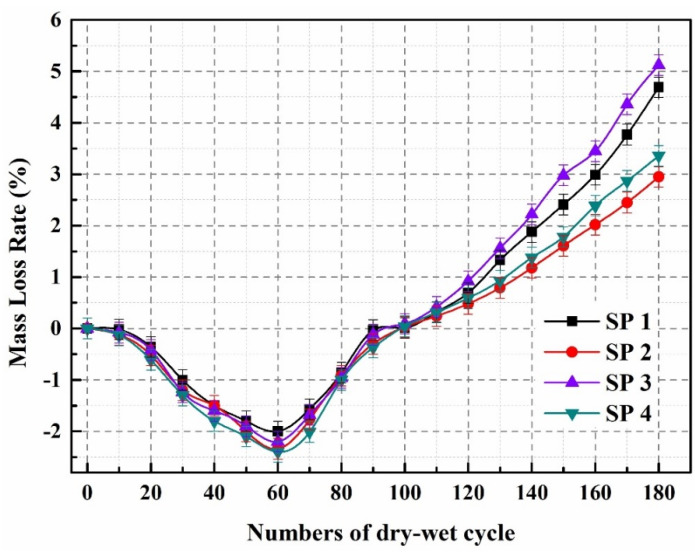
Mass loss rate of SP1–SP4.

The mass loss rate of SP1–SP4 during the sulfate dry–wet cycle is shown in Figure 2. The mass loss rate of SP1–SP4 showed a negative increase in the first 90 dry–wet cycles, due to the reaction between SO_4_^2−^ in Na_2_SO_4_ solution and the concrete matrix, resulting in the production of many expanding crystals and an increase in the specimen’s mass. The negative growth rate of mass loss in SP2 and SP4 is greater than that in SP1 and SP3. Because the particle size of microcapsules is much larger, when inserted into concrete, it leads to an increase in internal pores [41], making it easier for SO_4_^2−^ to penetrate into the concrete. Therefore, the negative growth in mass loss rate of SP2 and SP4 is more pronounced than that of SP1 and SP3. However, after 100 wet–dry cycles, the mass loss rate of all samples increased. After 180 dry–wet cycles, the mass loss rates of SP1, SP2, SP3, and SP4 were 4.69%, 2.95%, 5.12%, and 3.36%, respectively. Compared with SP1 and SP3, SP2 and SP4 have reduced their mass loss rates by 37.1% and 34.4%, respectively.

**Figure 3:**
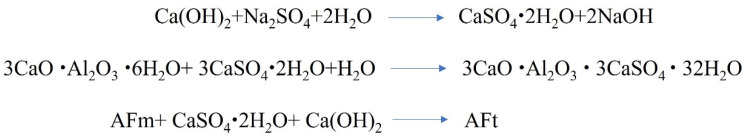
The mechanism of sulfate attack on concrete.

The observed phenomenon is attributed to the high concentration of SO_4_^2−^ in concrete, which leads to the crystallization and precipitation of gypsum dihydrate (CaSO_4_·2H_2_O) in cement stone. The conversion of Ca(OH)_2_ to gypsum leads to a doubling of the initial volume, resulting in the expansion and deterioration of concrete due to excessive internal tension. Meanwhile, SO_4_^2−^ interacts with Ca(OH)_2_, hydrated calcium aluminate, and AFm (monosulfide hydrated calcium sulfoaluminate) to form ettringite. Ettringite is a low solubility salt mineral, which contains a large amount of crystal water in its chemical composition. This increased the solid phase volume by 1.3 times. Ettringite precipitates as needle shaped crystals on the surface of the original hydrated calcium aluminate solid phase under mineral conditions. These crystals radiate in four directions, undergo growth, mutual compression, and generate significant internal strain, ultimately leading to the decomposition of the concrete structure. Figure 3 illustrates the key chemical reactions.

As the times of dry–wet cycles increase, internal fractures, peeling, and structural degradation will occur in concrete specimens, leading to rapid loss of concrete mass. When microcracks appear inside the concrete, the microcapsules will rupture due to the tension at the tip, releasing epoxy resin. Then, when it comes into contact with ethylenediamine, it will solidify, resulting in the filling of microcracks with repair products [42]. This reduces the mass loss rate of SP2 and SP4 and enhances their resistance to sulfates. Compared with SP2 (OPC concrete), SP4 (LC3 concrete) has a higher Al_2_O_3_ content in SCMs (Table 1). Al_2_O_3_ and sulfate can produce more ettringite (Figure 3), resulting in the higher expansion of SP4, increased porosity, promoted transport of SO_4_^2−^ in concrete, and allowing for repeated cycles. Therefore, the mass loss rate of SP4 is slightly higher than that of SP2.

### 2.2. Compressive Strength Loss Rate

Table 1 displays the compressive strength of SP1–SP4 after 28 days of curing. The compressive strength values for SP1, SP2, SP3, and SP4 are 56.9 MPa, 54.2 MPa, 54.8 MPa, and 52.1 MPa, respectively. Observing the experimental results of SP1 and SP3 reveals that substituting 50% of ordinary Portland cement with SCMs did not significantly alter the mechanical properties of concrete. In comparison to SP1 and SP3, the compressive strength of SP2 and SP4 decreased by 4.7% and 4.9%, respectively. This reduction is attributed to the larger particle size of microcapsules compared to other fine aggregates in the concrete, resulting in relatively lower mechanical performance. As an additive, microcapsules alter the gradation and compactness of the concrete, leading to a slight decrease in its mechanical properties [43].

**Figure 4:**
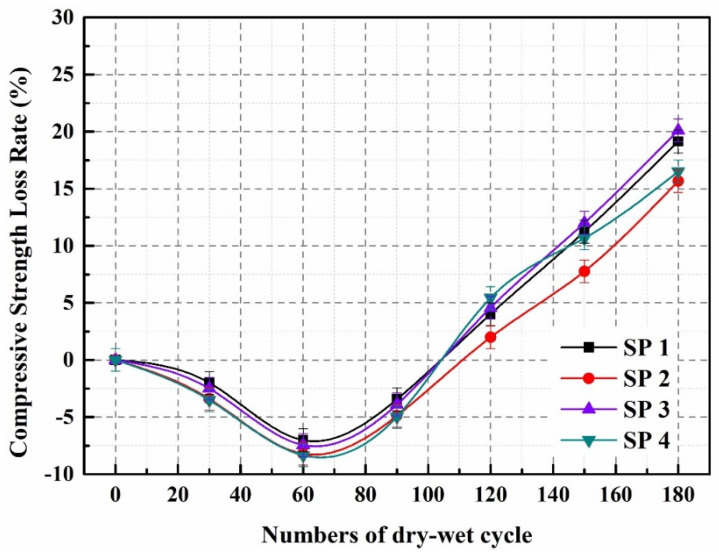
Compressive strength loss rate of SP1–SP4.

Figure 4 illustrates the rate of compressive strength loss in concrete during the sulfate dry–wet cycling process. Notably, all concrete specimens exhibit an initial negative growth trend in their compressive strength loss rate over the first 90 cycles. This trend can be attributed to the infiltration of sulfate ions (SO_4_^2−^) from the Na_2_SO_4_ solution, which reacts with hydration products to form expansive compounds like gypsum and ettringite. These reactions not only promote volume expansion but also contribute to a denser internal structure, ultimately enhancing the compressive strength of the concrete. During the dry–wet cycling, as specimens are dried, a small amount of sulfate crystals precipitates, further filling the micro-pores within the concrete and compacting its structure, which positively influences compressive strength. However, the larger particle size of the microcapsules compared to fine aggregates slightly compromises the overall compactness of the concrete matrix. This reduced density creates pathways for the sulfate ions to penetrate and diffuse more easily, leading to a more pronounced reduction in compressive strength for SP2 and SP4 than for SP1 and SP3.

During the wet–dry cycling process, the continual input of SO_4_^2−^ into the concrete reacts with the matrix, creating gypsum and ettringite and exerting internal expansion pressure in the concrete. During the drying process, sulfate crystals form within the concrete produce crystalline pressure. The number of dry–wet cycles increases the volume expansion and crystalline pressure in the concrete pores. When these forces surpass the matrix’s load-bearing limit, microcracks form inside, lowering compressive strength. Microcrack development enhances the pace at which SO_4_^2−^ penetrates the matrix, hastening the corrosive effects of sulfate on the concrete. As a result, after 100 dry–wet cycles, the concrete’s compressive strength decreases dramatically. After 180 dry–wet cycles, the compressive strength loss rates for SP1, SP2, SP3, and SP4 are 19.1%, 15.7%, 20.1%, and 16.5%, respectively. Figure 4 shows that the compressive strength loss rates of SP2 and SP4 after an equivalent number of dry–wet cycles are significantly lower than those of SP1 and SP3. These findings suggest that incorporating microcapsules into concrete might improve its resistance to sulfate corrosion. This improvement is related to the creation of fractures inside the concrete as a result of sulfate attack’s dry–wet cycles. Because of the force from fracture propagation, the microcapsules within the concrete break, releasing epoxy resin that combines with ethylenediamine to solidify and generate healing products. This procedure reduces damage to the concrete structure, resulting in a more restricted loss of compressive strength.

During the wet–dry cycling process, the continuous influx of SO_4_^2−^ into the concrete reacts with the matrix, leading to the formation of gypsum and ettringite. This reaction generates internal expansion pressures within the concrete. As the drying phase occurs, sulfate crystals precipitate, creating additional crystalline pressures. With each successive dry–wet cycle, both volume expansion and crystalline pressures within the concrete pores increase. When these internal forces exceed the load-bearing capacity of the matrix, microcracks begin to develop, compromising the compressive strength. The formation of microcracks not only diminishes the concrete’s structural integrity but also facilitates the penetration of sulfate ions into the matrix, accelerating the corrosive effects of sulfate attack. Consequently, after 100 dry–wet cycles, the concrete exhibits a substantial decline in compressive strength. Specifically, after 180 dry–wet cycles, the compressive strength loss rates for SP1, SP2, SP3, and SP4 are recorded at 19.1%, 15.7%, 20.1%, and 16.5%, respectively. Notably, Figure 4 reveals that SP2 and SP4 exhibit significantly lower compressive strength loss rates compared to SP1 and SP3 after an equivalent number of cycles. These observations indicate that the incorporation of microcapsules into concrete can enhance its resistance to sulfate corrosion. This enhancement can be attributed to the behavior of the microcapsules in response to the stresses induced by sulfate attack during the dry–wet cycles. As microcracks propagate, the internal pressures lead to the rupture of the microcapsules, which release epoxy resin that interacts with ethylenediamine to form solid healing products. This healing mechanism effectively mitigates structural damage, resulting in reduced compressive strength loss. Thus, the presence of microcapsules not only addresses the immediate impacts of sulfate corrosion but also actively contributes to the restoration of the concrete’s integrity, highlighting the potential of microcapsule technology in improving the durability of concrete structures exposed to harsh environmental conditions.

### 2.3. Corrosion Resistance Coefficients

**Figure 5:**
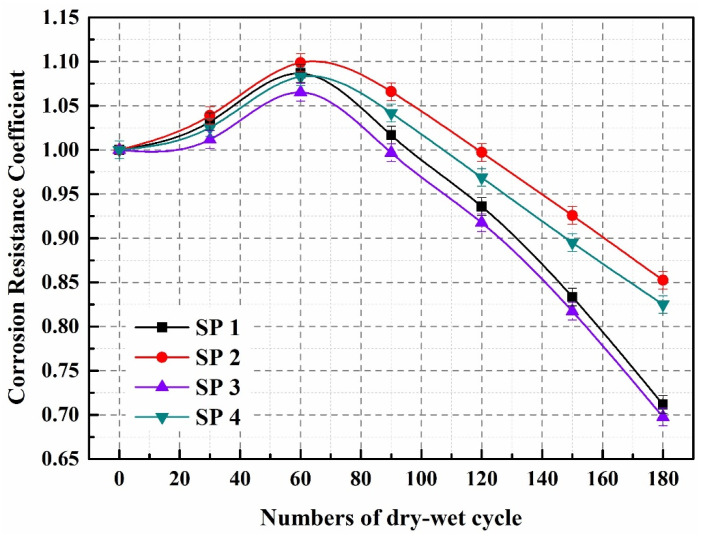
Corrosion resistance coefficient of SP1–SP4.

Figure 5 depicts the effect of sulfate dry–wet cycles on concrete corrosion resistance. In the initial 60 cycles, the corrosion resistance coefficients of SP1–SP4 all exceeded 1, with SP2 and SP4 exhibiting a more pronounced effect compared to SP1 and SP3. Because of the larger particle size of microcapsules, incorporating them into concrete raises the proportion of big holes marginally, allowing SO_4_^2−^ to penetrate into SP2 and SP4. Subsequently, SO_4_^2−^ reacts with the matrix, generating gypsum and ettringite, inducing expansive crystallization, and thus increasing the compressive strength of the concrete. After 60 cycles of dry–wet exposure, the corrosion resistance coefficients for SP1, SP2, SP3, and SP4 were 1.087, 1.099, 1.065, and 1.083, respectively.

The corrosion resistance coefficient of concrete notably decreased after 60 cycles of dry–wet exposure, indicating the material’s vulnerability to environmental stressors. However, the addition of microcapsules in SP2 and SP4 resulted in a less pronounced reduction in corrosion resistance compared to SP1 and SP3, suggesting that microcapsules play a crucial role in moderating the decline in concrete’s corrosion resistance. Specifically, after 180 dry–wet cycles, the corrosion resistance coefficients for SP1, SP2, SP3, and SP4 were measured at 0.712, 0.852, 0.698, and 0.823, respectively. As the number of dry–wet cycles increases, the accumulation of gypsum and ettringite within the concrete generates significant expansion stresses. When these stresses exceed the matrix’s load-bearing capacity, internal fractures begin to develop. These fissures not only compromise the structural integrity of the concrete but also create pathways that facilitate the transport of sulfate ions (SO_4_^2−^). This accelerated ingress of sulfates intensifies the corrosive processes affecting the concrete, leading to a rapid decline in its corrosion resistance coefficient.

According to the GB/T 50082-2009 standard [44], concrete’s internal structural integrity is typically compromised when the corrosion resistance coefficient falls below 0.75. After 180 cycles of dry–wet exposure, the corrosion resistance coefficients for SP1 and SP3 were recorded at 0.712 and 0.698, respectively, both below the critical threshold. This indicates significant deterioration in the structural integrity of these specimens. In contrast, SP2 and SP4 exhibited corrosion resistance coefficients of 0.852 and 0.823, respectively, both exceeding 0.76. This suggests that the incorporation of microcapsules effectively mitigates the effects of sulfate erosion. The underlying mechanism for this enhancement can be linked to the progressive formation of internal cracks within the concrete as the number of dry–wet cycles increases. As these cracks propagate, the stress concentrations at their tips lead to the rupture of the microcapsules’ outer shells. This rupture initiates the release of epoxy resin, which quickly reacts with ethylenediamine to form a binding agent that facilitates the self-healing process of the microcracks. This self-healing capability significantly enhances the concrete’s resistance to sulfate attack by effectively sealing the fissures that would otherwise serve as conduits for further sulfate infiltration [45,46]. Overall, the results underscore the critical role of microcapsule technology in improving the durability of concrete exposed to aggressive sulfate environments. By addressing both the initial and ongoing damage mechanisms, microcapsules provide a promising avenue for enhancing the longevity of concrete structures in challenging conditions, warranting further exploration into optimizing their composition and deployment for practical applications.

### 2.4. Pore Size Distribution

Pores with a diameter greater than 0.1 μm are generally considered harmful because they may allow the penetration of water, air, and other deleterious substances into the interior of concrete, leading to internal corrosion and degradation. These larger pores often result in stress concentration within the concrete, triggering the propagation of microcracks. When subjected to stress, these microcracks become initiation points for damage, reducing the overall strength of the concrete and posing a significant threat to its long-term durability.

**Figure 6:**
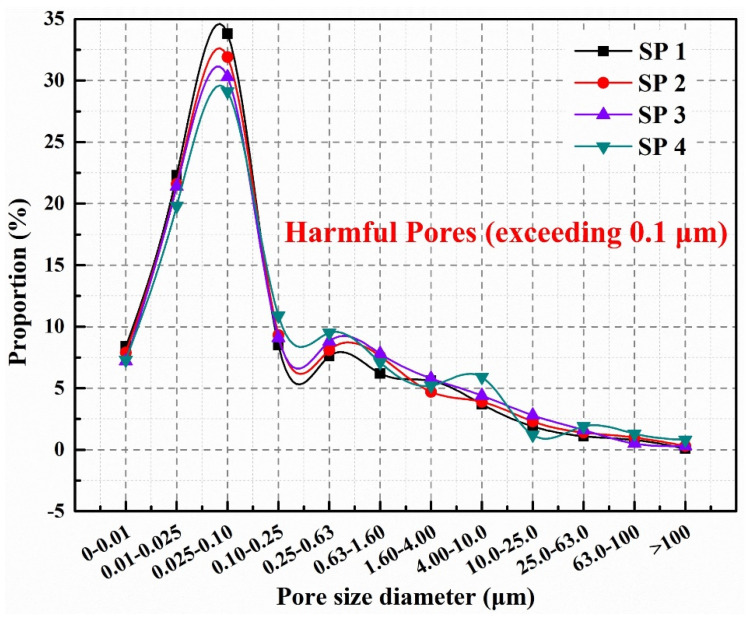
Pore size distribution of concretes.

We conducted a study based on the harmful pores distribution in the concrete to offer a more complete examination of the influence of microcapsules on the pore size proportion in concrete. From Figure 6, the proportion of harmful pores for SP1 and SP2 is 36.5% and 40.1%, respectively. This indicates that when using SCMs to partially replace cement, the pore size distribution in concrete is not significantly affected. For SP3 and SP4, the proportion of harmful pores is 38.6% and 43.8%, respectively. In comparison to SP1 and SP2, SP3 and SP4 show a slightly higher proportion of harmful pores. This is attributed to the role of microcapsules as fillers in concrete. However, their larger particle size compared to fine aggregates alters the grading of the concrete and increases the number of pores. Nevertheless, due to the limited mass of microcapsules used, constituting only 4% of the total mass of the binder materials, the proportion of harmful pores in the concrete increases by a little.

**Figure 7:**
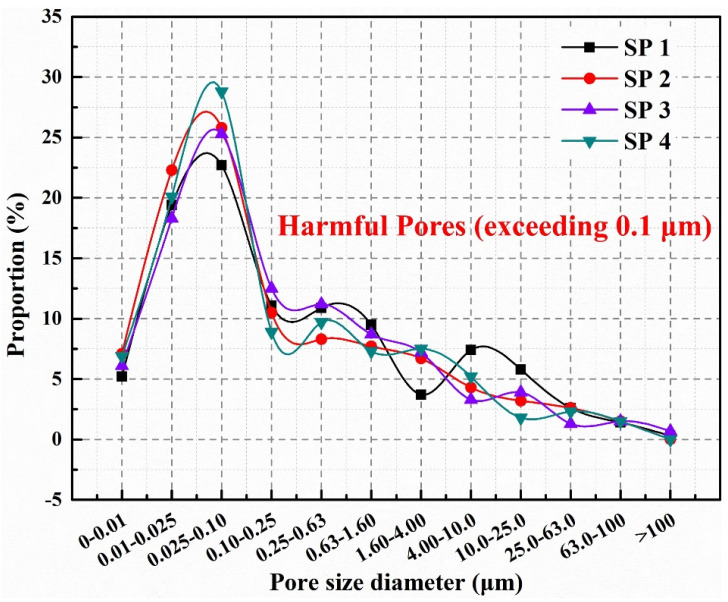
Pore size distribution of concretes after self-healing.

Figure 7 demonstrates the pore distribution of SP1–SP4 after 180 cycles of sulfate dry–wet exposure, following a 14-day self-healing period. Post self-healing, the proportions of harmful pores in SP1, SP2, SP3, and SP4 are 52.7%, 44.8%, 50.3%, and 44.2%, respectively. This demonstrates that after self-healing, microcapsule-containing concrete (SP2, SP4) has a lower percentage of harmful pores than control samples (SP1, SP3), which is attributed to sulfate infiltration into the dry–wet cycles of concretes, reacting with the cementitious matrix to form expansive crystals. The quantity of these expansive crystals rapidly increases with the number of dry–wet cycles, elevating the ratio of internal cracks and harmful pores in the concrete. SP1 and SP3, lacking microcapsules, are unable to rely on internal mechanisms for repair after the formation of cracks and harmful pores, resulting in a gradual loosening of the internal structure and a significant increase in the proportion of harmful pores. In contrast, SP2 and SP4, containing microcapsules, exhibit a unique self-healing mechanism. When microcracks occur inside the concrete, the shells of the microcapsules break owing to tension at the crack points, releasing epoxy resin, which interacts with ethylenediamine to generate a healing product, increasing the density of concretes and declining the proportion of harmful pores.

**Figure 8:**
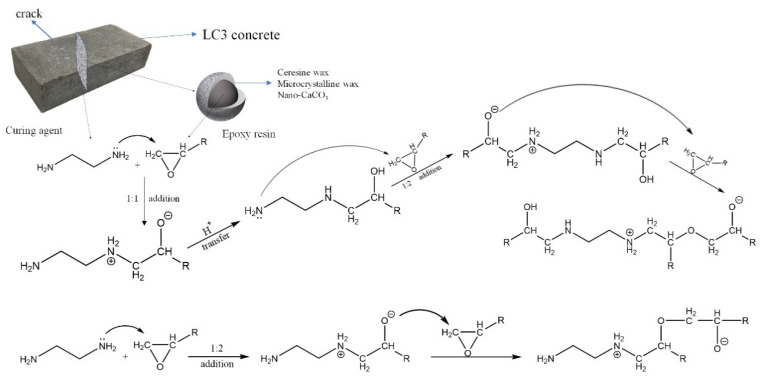
The chemical reaction of epoxy resin and ethylenediamine.

Consequently, concrete containing microcapsules demonstrates excellent self-healing capabilities. Figure 8 illustrates the reaction process between epoxy resin and ethylenediamine. After self-healing, the proportion of harmful pores in SP4 is restored to 99.1% of the initial value (28 days curing period), while for SP2, it is 89.5%. This may be attributed to the higher content of un-hydrated particles in the LC3 concrete (SP4) compared to the OPC concrete (SP2), indicating that LC3 concrete possesses superior self-healing capabilities. The synergistic effect with microcapsules further enhances the self-healing performance of LC3 concrete.

### 2.5. SEM Observation

**Figure 9:**
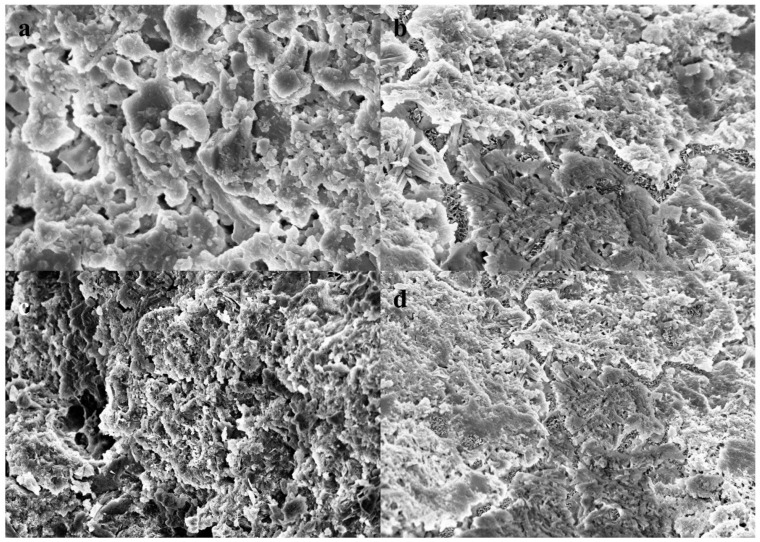
Microstructure of concretes after self-healing. (**a**) SP1, (**b**) SP2, (**c**) SP3, (**d**) SP4.

Figure 9 presents SEM images of concrete specimens subjected to 180 cycles of sulfate dry–wet exposure, followed by 14 days of self-healing. The microstructural differences among the various specimens are striking. In Figure 9a,c, SP1 and SP3 display numerous micropores and unhealed microcracks, indicative of a relatively loose internal structure. In contrast, the self-healed images of SP2 and SP4 (Figure 9b,d) reveal a mesh-like structure formed by healing products, suggesting a significantly more compact microstructure. The observed differences can be attributed to the continual penetration of sulfate ions into the concrete during the dry–wet cycles. This process generates expansive crystalline products through interactions with existing hydration products, leading to the formation of internal fissures. The incorporation of microcapsules plays a pivotal role in this context; as cracks develop, the microcapsules rupture at their tips, releasing epoxy resin. This resin rapidly reacts with ethylenediamine present in the concrete, resulting in the formation of healing products characterized by a mesh-like configuration, effectively sealing the cracks. SEM results further demonstrate that the presence of this mesh-like structure significantly enhances the microstructure of sulfate-affected concrete. The introduction of microcapsules not only bolsters the concrete’s resistance to sulfate attack but also endows it with remarkable self-healing capabilities. Moreover, the concrete structure depicted in Figure 9d appears denser than that in Figure 9b, a phenomenon attributed to the higher content of un-hydrated particles present in SP4. This, combined with the ongoing hydration processes and the action of microcapsules, contributes to a more robust self-healing response, resulting in a denser internal structure. These findings underscore the critical role of microcapsules in enhancing the durability and self-healing capacities of concrete under sulfate attack conditions. The ability of microcapsules to effectively repair and reinforce the internal microstructure not only mitigates the detrimental effects of sulfate exposure but also highlights their potential for improving the longevity of concrete structures in aggressive environments.

### 2.6. Compressive Strength Retention

Table 2 illustrates the compressive strength retention of concrete after 14 days of self-healing following 180 cycles of sulfate salt dry–wet exposure. The compressive strength retention for SP1 and SP3 is recorded at only 71.5% and 72.7%, while SP2 and SP4 demonstrate significantly higher values of 90.3% and 98.1%, respectively. This represents an impressive increase in compressive strength retention of 26.3% and 34.9% for SP2 and SP4 compared to SP1 and SP3. These findings underscore a substantial improvement in the resilience of concrete subjected to sulfate erosion.

The underlying reasons for this enhancement can be traced to the infiltration of SO_4_^2−^ from the Na_2_SO_4_ solution during the dry–wet cycles, which leads to the formation of expansive crystalline products such as gypsum and ettringite. This volumetric expansion exerts increasing tension on the internal pores and walls of the concrete, eventually causing microcracks to form when the expansion stress surpasses the matrix’s load-bearing capacity [47]. These microcracks facilitate the further penetration of sulfate ions, accelerating the deterioration process and resulting in a marked reduction in compressive strength retention.

However, the introduction of microcapsules into the concrete matrix mitigates these detrimental effects. As the microcracks develop, the stress at their tips causes the microcapsules’ outer shells to rupture, releasing epoxy resin. Upon contact with ethylenediamine within the concrete, this resin cures and forms a healing product that effectively seals the microcracks, restoring mechanical integrity [33]. This self-healing mechanism is particularly pronounced in SP4, which contains a greater quantity of un-hydrated particles. These particles not only contribute to the overall matrix density but also promote continued hydration, thereby enhancing the concrete’s self-healing capabilities.

Consequently, SP4 exhibits the highest compressive strength retention, demonstrating the synergistic effect of microcapsules and un-hydrated particles in combating sulfate-induced damage. These results highlight the potential of microcapsule technology as a promising solution for improving the durability and longevity of concrete structures exposed to aggressive environments, suggesting avenues for further research into optimizing their formulation and integration in concrete applications.

### 2.7. Chloride Ion Diffusion Coefficient

From Table 3, we can obtain the chloride ion diffusion coefficients of concrete at the age of 28 days. The chloride ion diffusion coefficients for SP1, SP2, SP3, and SP4 are 5.766 × 10^−12^ m^2^/s, 5.983 × 10^−12^ m^2^/s, 5.951 × 10^−12^ m^2^/s, and 6.132 × 10^−12^ m^2^/s, respectively. Compared to SP1 and SP3, the chloride ion diffusion coefficients of SP2 and SP4 have increased by 3.6% and 3.1%, respectively. This is attributed to the larger particle size of the microcapsules, altering the gradation of the concrete compared to the fine aggregates. The connecting surface between the microcapsules and the cement matrix may produce gaps, increasing internal pores and decreasing concrete compactness. Consequently, channels are created for the transmission of chloride ions, as a result of which the chloride ion diffusion coefficient increases and the concrete’s impermeability decreases [48]. This experimental result is consistent with the findings in Section 2.4. However, due to the microcapsule addition, its overall impact on the impermeability of the concrete is not substantial.

Table 3 also shows the preservation of concrete’s chloride ion diffusion coefficient following 180 cycles of sulfate dry–wet exposure, followed by a 14-day self-healing period. The retention of chloride ion diffusion coefficients for SP1, SP2, SP3, and SP4 are 58.6%, 76.2%, 61.5%, and 88.7%, respectively. The significantly higher retention of chloride ion diffusion coefficient of SP2 and SP4 compared to SP1 and SP3 indicate that microcapsules confer an excellent self-healing capability to the concrete. This is due to the creation of different expansive products in the concrete following 180 cycles of sulfate dry–wet exposure, which results in the generation of expansive stress and the formation of microcracks. The epoxy resin combines with the ethylenediamine to generate a finished product that plugs the microcracks, improving the retention of the chloride ion diffusion coefficient of concretes. Furthermore, the presence of certain un-hydrated particles in SP4 causes the concrete to hydrate, densifying the interior structure and increasing the retention of the chloride ion diffusion coefficient of SP4.

### 2.8. Ultrasonic Test

**Figure 10:**
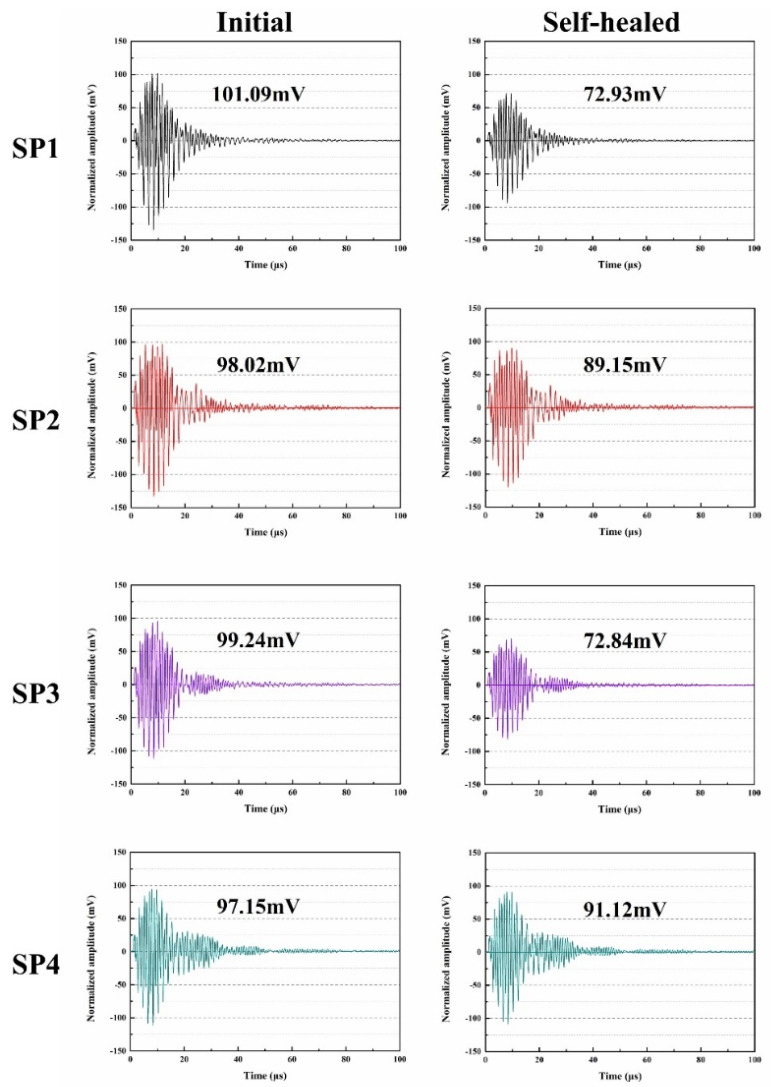
Ultrasonic waveform of concretes.

Figure 10 presents the ultrasonic waveforms of SP1 to SP4, including the initial state and the state after 14 days of self-healing. In media with different compaction densities, there are significant differences in the amplitude of ultrasonic wave propagation. Concrete, as a dense material, has tightly packed internal particle structures. This unique characteristic allows ultrasonic waves to maintain relatively large amplitudes when propagating through a compacting medium, as there is minimal energy loss. Additionally, concrete is an elastic material with a relatively high modulus of elasticity. Elastic materials exhibit high resilience, making them less prone to attenuation during wave propagation, thereby maintaining significant amplitudes. Furthermore, ultrasonic waves typically propagate in a straight-line path within concrete, showing minimal deviation or scattering, further contributing to the preservation of significant amplitudes [49].

However, the presence of cracks, voids, and other defects in concrete can affect the amplitude of ultrasonic wave propagation. Specifically, ultrasonic waves within cracks or voids undergo reflection and scattering processes, leading to partial reflection and scattering in different directions. These reflection and scattering processes disperse the energy of ultrasonic waves, resulting in a reduction in amplitude [50]. Therefore, in concrete, ultrasonic waves often exhibit larger amplitudes, but the presence of cracks and voids inside concrete can lead to amplitude reduction. These amplitude variations represent crucial information regarding ultrasonic non-destructive testing and are used to assess the integrity and quality of concrete structures. The initial maximum amplitudes of SP1, SP2, SP3, and SP4 were 101.09 mV, 98.02 mV, 99.24 mV, and 97.15 mV, respectively. Compared to SP1 and SP3, SP2 and SP4 exhibit a reduction of 3.1% and 2.1% in maximum amplitude. As shown in Figure 6 in Section 2.4, the harmful pore ratios of SP2 and SP4 are higher than those of SP1 and SP3, suggesting that they contain more internal faults and a looser structure, resulting in a lower maximum amplitude of ultrasonic waves transmitted through these specimens.

The maximum amplitudes measured after 14 days of self-healing for SP1, SP2, SP3, and SP4 are 72.93 mV, 89.15 mV, 72.84 mV, and 91.12 mV, respectively. These results indicate that the incorporation of microcapsules significantly enhances the ability of concrete specimens to recover their maximum amplitude following sulfate erosion. The underlying mechanism for this recovery can be attributed to the expansion stresses generated by the formation of gypsum and ettringite, which lead to the development of microcracks within the concrete. This damage results in a notable decrease in the maximum amplitude for SP1 and SP3.

In contrast, when microcracks occur in SP2 and SP4, they tend to propagate toward the embedded microcapsules. Under stress conditions, the shell material of these microcapsules ruptures, releasing epoxy resin. This resin then reacts with the ethylenediamine pre-mixed within the concrete to form a healing product that effectively seals the microcracks. Consequently, this process not only reduces internal flaws within the concrete but also enhances its compaction and increases the measured maximum amplitude.

Moreover, SP4’s composition includes un-hydrated particles, which, alongside the action of microcapsules, further bolster the self-healing capabilities of the concrete through continued hydration. As a result, SP4 demonstrates the highest maximum amplitude among the specimens, achieving a recovery that closely approaches its initial value—restored to 93.8%. This impressive restoration highlights the synergistic effects of microcapsules and un-hydrated particles in reinforcing concrete’s durability and self-healing properties, suggesting that further optimization of these components could lead to even greater enhancements in performance under sulfate exposure conditions. These findings underscore the potential of microcapsule technology as a transformative approach in concrete design aimed at improving long-term structural integrity.

**Figure 11:**
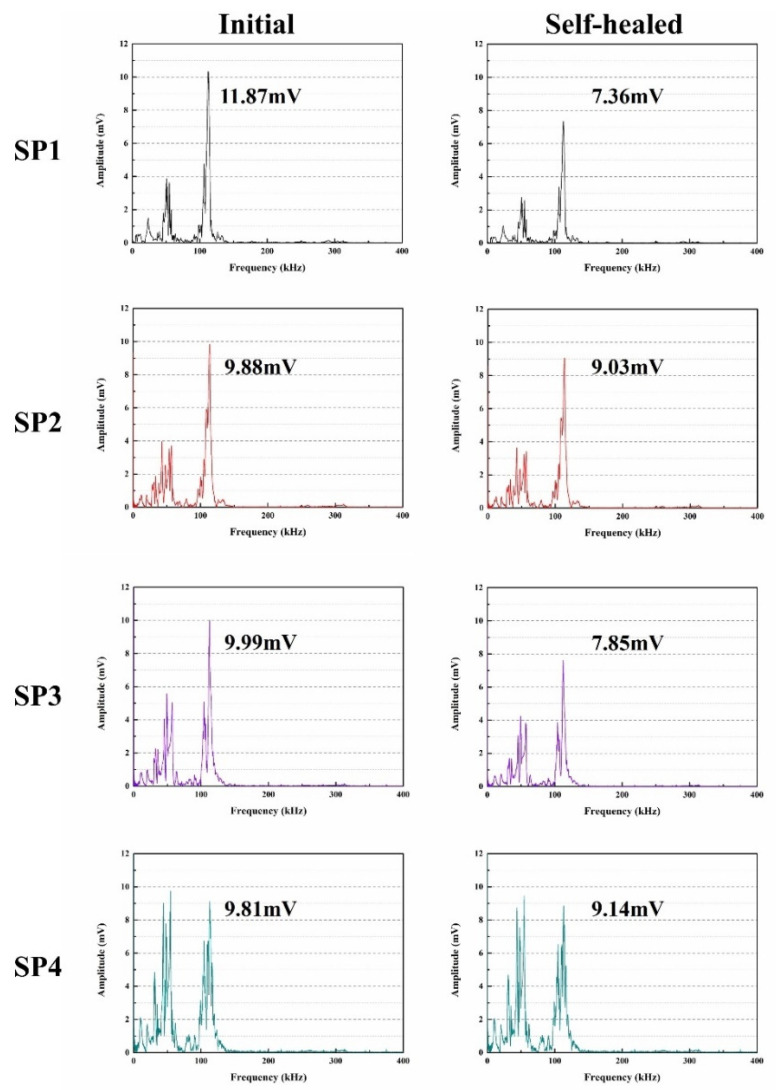
Ultrasonic frequency of concretes.

The Gaussian function’s Fast Fourier Transform (FFT) can be used to evaluate changes in ultrasonic waves and produce more precise experimental results [51]. As shown in Figure 10, the ultrasonic waves of SP1, SP2, SP3, and SP4 underwent FFT transformation to study their frequency domain curves in both the initial and self-healing states. The main frequency of the experiment was close to the resonance frequency of 107 kHz. From Figure 11, it can be observed that the initial main frequency’s maximum amplitudes for SP1, SP2, SP3, and SP4 were 11.87 mV, 9.88 mV, 9.99 mV, and 9.81 mV, respectively. The initial maximum amplitude of the main frequency in concrete is connected to the initial ratio of harmful pores in the concrete, as explained in Section 2.4. Compared to SP1 and SP3, the initial ultrasonic frequency of SP2 and SP4 is lower, indicating a higher proportion of harmful voids. This is because introducing microcapsules into concrete may alter the particle size distribution [52], causing holes or faults at the binding surface of microcapsules and cement matrix, leading to signal loss during the propagation of ultrasonic waves, ultimately reducing the maximum amplitude of the concrete’s main frequency.

Figure 11 illustrates the maximum frequency amplitudes after self-healing for SP1, SP2, SP3, and SP4, measuring 7.36 mV, 9.03 mV, 7.85 mV, and 9.14 mV, respectively. In comparison to the initial state, the maximum frequency amplitudes of SP1 and SP3 significantly decreased. This is because sulfate attack induces the formation of crystalline products in the concrete, such as gypsum and ettringite, which generate expansion stress and microcracks. Because concrete is unable to endure these loads, the ultrasonic signal weakens, resulting in a decrease in the maximum amplitude for SP1 and SP3. The FFT transformation converts the maximum frequency amplitude from its peak value to the maximum magnitude, producing a numerical trend similar to the maximum magnitude. During the concrete preparation process, microcapsules (microcrystalline wax, ceresine wax, and nano-CaCO_3_ coated with epoxy resin) were introduced. As inert materials, microcrystalline wax and ceresine wax are not susceptible to corrosion in the high alkaline environment of concrete. Sulfate ions do not promote the rupture of microcapsule shells in dry–wet cycle experiments, successfully retaining the epoxy resin within the microcapsules. When microcracks occur in the cement matrix as a result of the cement’s failure to tolerate gypsum and ettringite expansion pressures, the microcapsules rupture at the tips of the cracks, releasing epoxy resin in contact with ethylenediamine, forming a healing product that fills the cracks. This prevents the further extension of microcracks, mitigating potential damage to the concrete structure and enhancing concrete durability. Consequently, the maximum frequency amplitudes for SP2 and SP4 noticeably recover after self-healing. A few of un-hydrated particles exist in SP4, and after continuous hydration, they, together with the microcapsules, enhance the self-healing performance of concretes.

### 2.9. Healing Rates

**Figure 12:**
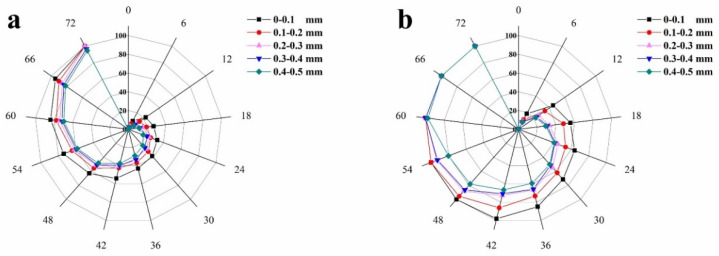
The surface crack healing rates of concretes: (**a**) SP2, (**b**) SP4.

The surface crack healing rates of SP2 and SP4 are shown in Figure 12. Comparing Figure 12a and Figure 12b, for surface cracks with initial widths in the range of 0–0.1 mm, SP2 achieved self-heal within 72 h, while SP4 completed the heal in just 48 h. As the crack width increased to 0.1–0.2 mm, SP2 required 72 h for self-heal, whereas SP4 only needed 54 h. Within 72 h, the maximum crack width that SP2 could heal was in the range of 0.2–0.3 mm. In comparison to SP2, SP4 could self-heal cracks with larger initial widths, even when the crack width reached 0.4–0.5 mm, SP4 only needed 66 h to complete the crack self-heal. By comparing Figure 12a and Figure 12b, it is evident that even with the same initial surface crack width, SP4 exhibits shorter self-heal time and higher heal efficiency. This significant improvement can be attributed to the design and performance characteristics of incorporating microcapsules into concrete.

In a detailed examination of the performance of these microcapsules, we observed that cracking is a critical triggering factor in the self-heal process. Concrete cracking leads to the formation of cracks. In the microcapsules, there exists a self-triggering mechanism related to the stress in the concrete cracks. The extension of cracks generates stress, which can cause the outer shell of the microcapsules to rupture, thereby releasing the encapsulated epoxy resin. The epoxy resin can react and heal with ethylenediamine, effectively filling the cracks. In comparison to SP2, SP4 contains some partially un-hydrated particles (originating from SCMs), which continuously hydrate and enhance the self-heal capability of concrete. The synergistic effect of these un-hydrated particles and microcapsules not only enhances the self-heal ability of concrete but also has the potential to positively impact the maintenance and durability of concrete structures.

## 3. Materials and Methods

### 3.1. Materials

Microcrystalline wax, ceresine wax, anhydrous sodium sulfate (Na_2_SO_4_), sodium chloride (NaCl), and nano-CaCO_3_ were purchased from Shanghai Haohong Biomedical Co., Ltd. (Shanghai, China). Perfluoro-tributylamine (PFTBA), epoxy resin, N, N-dimethylformamide (DMF), and ethylenediamine (EDA) were supplied by Wuhan Huaxiang Kejie Co., Ltd. (Wuhan, China). Ordinary Portland cement (OPC, PO 42.5) was supplied by China Resources Cement Co., Ltd. (Shenzhen, China). The method of obtaining calcined clay has been mentioned in our previous work [53]. Gypsum was purchased from Changde Hongguan Gypsum Environmental Protection Material Technology Co., Ltd. (Changde, China). Limestone powder was supplied by Hebei Jiegui Mineral Products Co., Ltd. (Shijiazhuang, China). The summary of chemical composition is shown in Table 4. Standard sand was bought from Chongqing Pinhetai Building Materials Co., Ltd. (Chongqing, China).

### 3.2. Microcapsule Preparation

**Figure 13:**
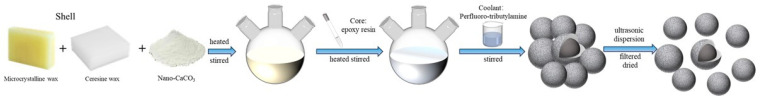
Schematic of the microcapsule preparation process.

**Figure 14:**
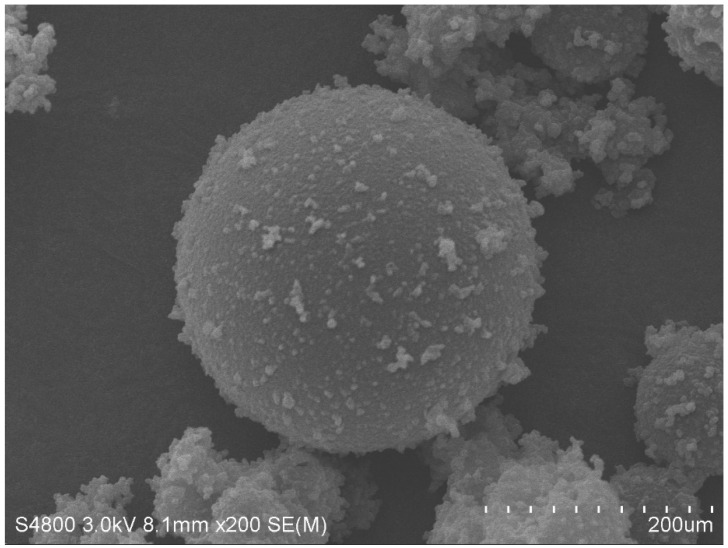
SEM image of the microcapsule.

According to prior investigations, microcapsules were created using the melt condensation process [54] as follows: Commencing with the amalgamation of microcrystalline wax and ceresine wax into a flask, the temperature is set to 115 °C. Following the complete melting of the microcrystalline wax and paraffin wax, stirring is conducted at a rate of 1000 rpm for one hour under continued heating. Subsequently, nano-CaCO_3_ is introduced into the flask, and the mixture is stirred and heated for an additional 30 min. Then, epoxy resin is added to the flask, and the mixture is subjected to stirring and heating for a duration of 2 h. Ceasing the heating process, the stirring speed is maintained while perfluorotributylamine (PFTBA) is introduced into the flask, inducing cooling to facilitate the formation of microcapsules. Finally, the microcapsules are filtered and subjected to a drying process. The detailed procedure for the preparation of microcapsules can be found in Figure 13. Table 5 provides a description of the various indicators of microcapsules. The microcapsules are depicted in Figure 14.

### 3.3. Concrete Preparation

According to the specifications of GB/T 50082-2009, the preparation and curing process of concrete specimens is outlined as follows: Prior to commencing mixing in the concrete mixer, a pre-mixing machine wetting procedure is conducted. Subsequently, pre-weighed cement, SCMs, aggregate, fly ash, mineral powder, and standard sand are introduced into the mixer and stirred for 90 s. Following this, water, water-reducing agent, and microcapsules are added to the blender and mixed for an additional 90 s. The concrete is then poured into molds of dimensions 100 × 100 × 100 mm^3^ and ϕ 100 mm × 50 mm, respectively. Vibrations are applied to the concrete on a vibrating table until surface bleeding ceases, after which excess slurry is removed using a spatula. Upon completion of molding, waterproof membranes are immediately attached to the concrete’s surface, and the specimens are moved to a curing room (20 ± 2 °C, 95% RH). After 24 h, demolding is carried out. The specific mix proportions for this experiment are presented in Table 6.

### 3.4. Dry–Wet Cycle of Sulfate

**Figure 15:**
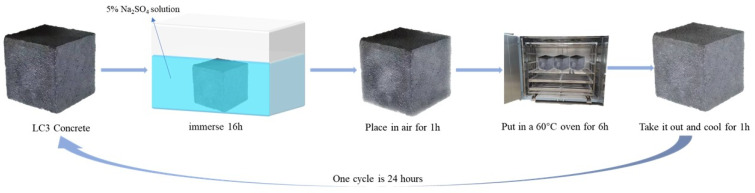
Sulfate dry–wet cycles experiment of concretes.

Concrete specimens that have undergone standard curing for 26 days are exposed to a 48 h exposure in an oven set at 60 °C, according to the GB/T 50082-2009 sulfate erosion test. Subsequently, specimens are taken from the oven and allowed to reach room temperature in the laboratory. Prior to the commencement of the experiment, the concrete specimens are categorized into two groups. The first group is placed in plastic containers containing a 5% concentration of sodium sulfate solution and sealed. The second group is cured continuously in a standard curing room. The following is the precise protocol for the sulfate dry–wet cycle experiment: Immerse the concrete for 16 h in a sodium sulfate solution, then remove and air-dry for 1 h. After that, place the concrete specimens in a 60 °C oven for 6 h, then remove and cool for 1 h. One cycle in this experiment lasts 24 h. The sodium sulfate solution is refilled every 7 cycles, for a total of 180 dry–wet cycles. Figure 15 illustrates the flowchart of the dry–wet cycle of sulfate.

#### 3.4.1. Mass Loss

Initiate the initial mass testing of 28-day-old concrete specimens. Subsequently, conduct real-time mass measurements for specimens after every 10 dry–wet cycles. Utilize three samples for each test to acquire the averaged data. Equation (1) is used to determine the specimens’ mass loss rate following each dry–wet cycle [55].

(1) $$\Delta M_{n}=\frac{M_{0}-M_{n}}{M_{0}}\times 100\%$$

In the equation, ΔM_n_ represents the mass loss rate (%) of concrete after n times of dry–wet cycles, M_0_ denotes the initial mass of the concrete (g), and M_n_ signifies the mass of the concrete after n times of dry–wet cycles (g).

#### 3.4.2. Compressive Strength Loss

Upon the completion of the 28-day curing period for the concrete specimens, retrieve the samples from either the oven or curing chamber, clean the surfaces thoroughly, and conduct initial compressive strength testing. Subsequently, measure the real-time compressive strength of specimens. The concrete’s loss rate of compressive strength decrease following sulfate dry–wet cycles is determined using Equation (2).

(2) $$\Delta f_{n}=\frac{f_{0}-f_{n}}{f_{0}}\times 100\%$$

In the equation, Δf_n_ represents the compressive strength loss rate (%) of concrete after n times of dry–wet cycles, f_0_ signifies the initial compressive strength of the concrete (MPa), and f_n_ denotes the compressive strength of the concrete after n times of dry–wet cycles (MPa).

#### 3.4.3. Corrosion Resistance Coefficient

Concrete’s compressive strength changes during cycles of sulfate-induced dry–wet exposure. Concrete’s sulfate resistance is expressed as the corrosion resistance coefficient (K), which is determined by applying Equation (3). This coefficient is a quantitative indicator of the concrete’s resistance to sulfate-induced deterioration, representing variations in compressive strength across the dry–wet cycles [55].

(3) $$K=\frac{f_{n}}{f_{i}}\times 100$$

K represents the corrosion resistance coefficient of concrete specimens, where f_n_ denotes the compressive strength of the concrete specimens after n dry–wet cycles (MPa), and f_i_ represents the compressive strength of standard-cured specimens of the same age as the dry–wet cycle specimens (MPa).

### 3.5. Self-Healing of Concrete after Sulfate Attack

#### 3.5.1. Pore Size Distribution

Using nuclear magnetic resonance testing, the principles of molecular resonance occurring in the water-filled pores of concrete in the presence of a magnetic field are employed to determine the pore size distribution. This phenomenon induces energy exchange and relaxation behavior, with the relaxation time being proportional to the pore size. Variations in pore size are reflected through changes in relaxation time. Following concrete specimen compressive strength testing at 28 days and 14 days of self-healing, approximately 1 cm^3^ samples are randomly extracted from concrete fragments. These samples are then cleaned of surface dust, dried in a 120 °C oven for 6 h, and subsequently saturated with water in a vacuum saturation device. Saturation is achieved by applying a continuous pressure of 30 MPa for 10 h in the saturation device. After completing the saturation experiment, the volume and nuclear magnetic resonance T_2_ spectrum of the samples are measured using the Suzhou Newmax Analytical Instruments Co., Ltd. (Suzhou, China) MicroMR23-025V nuclear magnetic resonance spectrometer. The resonance frequency is set at 23.40 MHz, with temperature control maintained at 32.00 ± 0.02 °C. Pore size distribution is calculated based on Equation (4) [55].

(4) $$\frac{1}{T_{2}}=\rho {\left(\frac{S}{V}\right)}_{\mathrm{pore}}$$

where T_2_ is the relaxation time of water in the pore (ms), ρ is the surface relaxation rate (70 μm/ms), and (S/V) pore is the pore surface area to volume ratio.

#### 3.5.2. Microstructure

Observations of microscopic morphology were made on several sets of concrete specimens exposed to 180 dry–wet cycles. A field emission scanning electron microscope (SEM) was used in the experiment (Zeiss Ultra Plus, Oberkochen, Baden-Württemberg, Germany). The voltage was 5 kV with a 10,000 times magnification. The specimens were immersed in absolute ethanol and dried before testing.

#### 3.5.3. Mechanical Properties

Following 180 cycles of dry–wet, specimens were removed and subjected to a 14-day self-healing procedure in the laboratory. The compressive strength was then determined. Each compressive strength retention test group consisted of three specimens. Furthermore, Equation (5) was applied to compute the compressive strength retention for concrete incorporating microcapsules.

(5) $$\eta_{fe}=\frac{f_{\mathrm{ni}}}{f_{0}}\times 100\%$$

In the equation, η_fe_ represents the compressive strength retention (%) of concrete, f_0_ stands for the initial compressive strength of the concrete (MPa), and f_ni_ represents the compressive strength of concrete after inducing self-healing through 180 dry–wet cycles and subsequent curing (MPa).

#### 3.5.4. Permeability

After 180 cycles of dry–wet exposure, specimens were put in the laboratory for a 14-day self-healing period, during which the chloride ion diffusion coefficients of each specimen were calculated. Three concrete specimens were evaluated for each experimental group, and the average values were utilized for computations. The retention of chloride ion diffusion coefficients for the concrete specimens can be obtained using Equation (6).

(6) $$\theta_{n}=\frac{\theta_{0}}{\theta_{t}}\times 100\%$$

In the equation, θ_n_ represents the retention (%) of chloride ion diffusion coefficient, θ0 denotes the chloride ion diffusion coefficient of concrete specimens after standard 28-day curing (10^−12^ m^2^/s), and θ_t_ signifies the chloride ion diffusion coefficient of sulfate corroded concrete specimens after self-healing (10^−12^ m^2^/s).

#### 3.5.5. Ultrasonic Test

The practical utility of concrete relies on critical attributes such as mechanical properties, permeability, and compactness. Failures related to the compromise of these attributes have resulted in significant societal and individual losses. While several studies have been conducted to explore the macro-mechanical properties and permeability of concrete, many of these investigations have resulted in the destruction of specimens [33,34]. In real-world applications, it is critical to analyze and monitor various structural characteristics non-destructively to assure their integrity. Therefore, the application of nondestructive testing (NDT) methods holds paramount significance. Ultrasonic testing emerges as a rapidly advancing and widely adopted NDT technique, playing a crucial role in the field of nondestructive evaluations [56].

The generation and transmission of ultrasonic waves were achieved using an ultrasonic wave generator (AFG3022C, Tektronix Co., Ltd., Beaverton, OR, USA). Subsequently, the ultrasonic waves were received and characterized using an oscilloscope (MDO 3024, Tektronix Co., Ltd.). The frequency of the ultrasonic waves was maintained consistently at 107 kHz, with the voltage precisely regulated within the range of ±5 V. During the data collection process, ultrasonic waves were emitted by a radial piezoelectric ultrasonic transducer. These transducers were conventionally positioned on both sides of the specimen and secured in place with petroleum jelly. Throughout the testing phase, an automatic connection was established between the data and images through a computer interface. The assessment of concrete specimens took place after undergoing 180 dry–wet cycles and a 14-day self-healing period.

#### 3.5.6. Cracks Self-Healing

**Figure 16:**
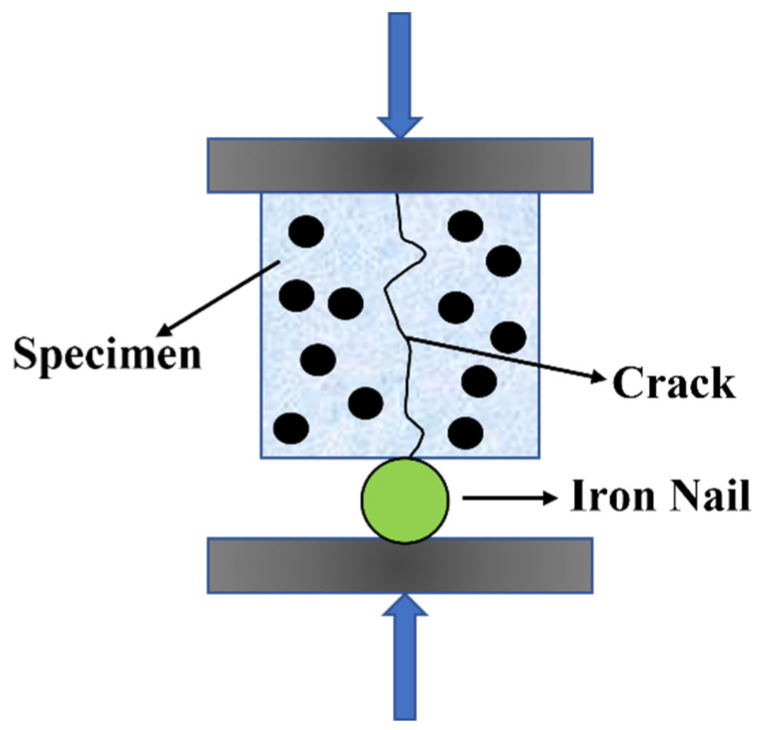
Schematic diagram of the concretes’ splitting test.

Cylindrical concrete specimens, measuring Φ 100 mm × 200 mm at 28 days of age, were meticulously extracted from the standard curing room. These specimens were subsequently subdivided into 50 mm height samples. To induce controlled crack initiation during the splitting process, the circumferential surface of the cylindrical specimens was gently encircled with adhesive tape, preventing uncontrolled splitting. The prepared specimens were then positioned onto a fully automated compression testing machine. Applying a constant loading rate ranging from 0.3 to 0.5 kN/s, a compressive load was progressively applied to each specimen. Figure 16 is a schematic diagram. Loading was immediately halted upon the appearance of cracks in the concrete, and the specific location requiring analysis was duly marked. A visualization software was employed to quantify the number of pixels in the crack image of pre-cracked specimens, enabling the identification of crack regions with varying self-heal times. The calculation of surface crack healing rates was conducted according to Equation (7).

(7) $$\delta =-\frac{\delta_{t}-\delta_{i}}{\delta_{i}}\times 100\%$$

where *δ* is the healing rate of crack (%), *δ_i_* is the initial number of pixels of crack, *δ_t_* is the number of pixels of crack after t h of self-healing.

## 4. Conclusions

To reduce carbon emissions and energy consumption in the production and use of building materials, this study replaced 50% of OPC with limestone, calcined clay, and gypsum in the preparation of LC3 concrete, and introduced thermoplastic microcapsules to enhance its resistance to sulfate attack and self-healing capabilities. The experiments demonstrated that the addition of microcapsules significantly improved the concrete’s resistance to erosion. After 180 wet–dry cycles, SP4 maintained low mass loss and high compressive strength, exhibiting strong self-healing properties by repairing cracks with an initial width of 0.4–0.5 mm within 66 h. Microstructural analysis revealed that the presence of microcapsules facilitated the formation of healing products, further enhancing the durability of the material. These findings suggest that thermoplastic microcapsule technology can extend the lifespan of concrete structures. It is recommended that future research focus on the long-term stability of microcapsules in various corrosive environments, optimize microcapsule formulations to improve healing efficiency, and conduct large-scale testing in real-world applications to assess its economic feasibility and practical potential.

## Figures and Tables

**Table 1:** Compressive strengths of the concrete.

Specimen	Compressive Strength (28-Day Age)
SP1	56.9 MPa
SP2	54.2 MPa
SP3	54.8 MPa
SP4	52.1 MPa

**Table 2:** Concrete compressive strength retention.

Specimen	Compressive Strength Retention
SP1	71.5%
SP2	90.3%
SP3	72.7%
SP4	98.1%

**Table 3:** Chloride ion diffusion coefficient and retention of chloride ion diffusion coefficient of concretes.

Specimen	Chloride Ion Diffusion Coefficient	Retention of Chloride Ion Diffusion Coefficient
SP1	5.766 × 10^−12^ m^2^/s	58.6%
SP2	5.983 × 10^−12^ m^2^/s	76.2%
SP3	5.951 × 10^−12^ m^2^/s	61.5%
SP4	6.132 × 10^−12^ m^2^/s	88.7%

**Table 4:** Chemical composition of cementitious binders.

	OPC	Limestone	Gypsum	Calcined Clay
CaO	60.23%	55.77%	36.87%	0.27%
SiO_2_	21.69%	0.98%	13.66%	62.77%
MgO	2.65%	1.01%	3.79%	0.62%
Al_2_O_3_	6.06%	0.32%	5.75%	26.21%
Fe_2_O_3_	4.08%	0.08%	2.36%	5.98%
K_2_O	0.89%	-	-	3.22%
SO_3_	2.33%	0.06%	36.87%	-
Na_2_O	0.09%	-	-	-
TiO_2_	0.32%	-	-	0.81%
LOI	1.66%	37.69%	0.25%	0.69%

**Table 5:** Various indicators of microcapsules.

Composite (Weight Percentage)	Average Particle Size	Core Content	Surface Area	Elastic Modulus	Hardness	Weight Loss Rate in 60 Days
microcrystalline wax (20%) ceresine wax (20%) nano-CaCO_3_ (3%) epoxy resin (53%)	215 μm	81.2%	145 m^2^/g	1.99 GPa	71.99 MPa	1.5%

**Table 6:** Specific mix proportions of concretes.

Specimen		SP1	SP2	SP3	SP4
Cement		340	340	170	170
	Calcined Clay	0	0	102	102
SCMs	Limestone	0	0	51	51
	Gypsum	0	0	17	17
Fly Ash		40	40	40	40
GGBS		60	60	60	60
Sand		682	682	682	682
Stone		1080	1080	1080	1080
Superplasticizer		4.4	4.4	4.4	4.4
Water		154	154	154	154
Microcapsule		0	10.2	0	10.2

## Data Availability

Data are contained within the article.
